# First Metatarsophalangeal Joint Replacement in a Patient With Monostotic Fibrous Dysplasia and Associated Metatarsophalangeal Arthritis

**DOI:** 10.7759/cureus.46541

**Published:** 2023-10-05

**Authors:** Neil Limaye, Mohit Sethi, Brijesh Ayyaswamy

**Affiliations:** 1 Department of Trauma and Orthopaedics, North Tees and Hartlepool NHS Foundation Trust, Stockton-on-Tees, GBR

**Keywords:** patient-centered outcomes research, arthritis, first metatarsal, arthroplasty, fibrous dysplasia (fd)

## Abstract

Fibrous dysplasia (FD) is a benign disorder characterized by the replacement of normal bone tissue with fibrous connective tissue, making the bone more susceptible to fractures and increasing the risk of developing degenerative arthritis in multiple joints. We present an unusual case of monostotic FD affecting the first metatarsal, accompanied by metatarsophalangeal (MTP) arthritis, which caused difficulties in walking, pain, and reduced quality of life. The patient underwent the first MTP joint replacement using the mobile bearing ROTOglide™ first MTP joint replacement system; the use of this specific implant for this indication appears to be a novel aspect in the existing literature. Following the operation, the patient returned to normal activities, experiencing improvements in pain, walking, and quality of life, thus demonstrating excellent outcomes.

## Introduction

Fibrous dysplasia (FD) is a condition characterized by the replacement of normal bone tissue with fibrous connective tissue secondary to altered osteogenesis [1]. This leads to the weakening of the bone and increases its susceptibility to fractures and arthritis. FD is a non-inherited condition, possibly linked to mutations in the GNAS1 gene [2]. It can affect a single bone (monostotic disease (MFD)), multiple bones (polyostotic disease (PFD)), or skull and facial bones only (craniofacial fibrous dysplasia (CFD)) [2]. MFD accounts for 75-80% of cases [3]. Some reports have described the general affection of feet and ankles [4,5,6]. However, no literature describes first metatarsophalangeal arthritis or its treatment in a patient with FD of the first metatarsal. This is the first report describing the use of a modern joint replacement technique in the foot affected with FD. The implant used is the first metatarsophalangeal joint replacement system intended for use in metatarsophalangeal joints affected by osteoarthritis when other conservative measures have failed [7,8]. While there is sufficient literature to support its use in arthritic joints, this constitutes the first report describing its application in MFD [7,8]. In this case report, we will look at symptomatic MFD affecting the first metatarsal with first metatarsophalangeal joint arthritis, requiring joint replacement using a ROTOglide™ first MTP joint replacement system.

## Case presentation

**Figure 1:**
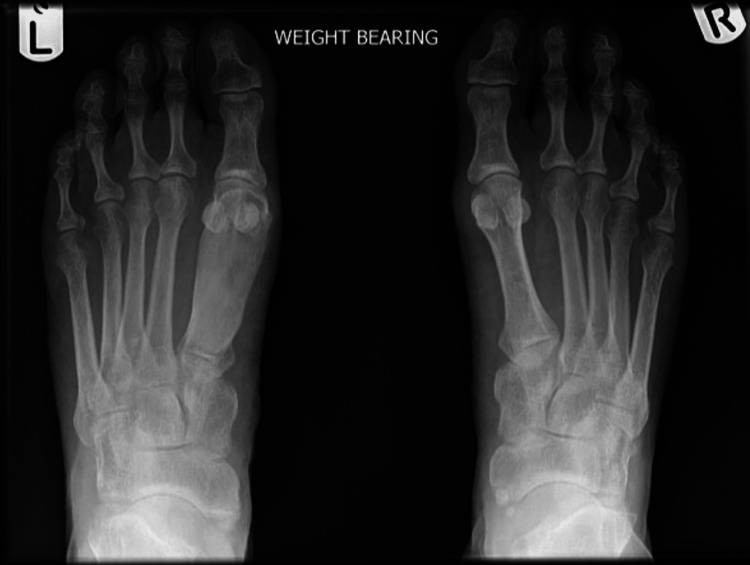
Anteroposterior pre-operative X-ray showing arthritic changes in the left metatarsophalangeal (MTP) joint.

The patient, a 57-year-old female, initially presented to her GP with a sudden onset of pain and swelling in her left toe's metatarsophalangeal joint, significantly impeding her walking and affecting her quality of life. There was no history of trauma. Clinical examination revealed a swollen first toe that was tender to touch, with no distal neurovascular deficit. Her past medical history included a duodenal ulcer, hay fever, and irritable bowel syndrome (IBS). Medications included cetirizine, mebeverine, and, more recently, hormone replacement therapy. Her social history involved smoking 15 cigarettes a day and occasional alcohol use. The patient had experienced symptoms for three years prior to consulting an orthopaedic surgeon. An initial X-ray of her foot was performed, which revealed an expanded first metatarsal with ground glass appearance and osteoarthritic changes of the first metatarsophalangeal joint (Figure 1). She was referred to the orthopaedic specialist services, who performed another X-ray and an MRI of her foot. Clinical examination by an orthopaedic surgeon and thorough radiological analysis by a musculoskeletal radiologist yielded a primary differential of monostotic fibrous dysplasia. Following this, an open biopsy of this lesion was taken, which confirmed the diagnosis. This was taken from the intra-medullary bone from the proximal first metatarsal and the head of the first metatarsal during the preparation of the joint prior to implanting the ROTOglide™ prosthesis. Initially, she was managed conservatively with analgesia, a stiff sole under the big toe, and observation since her symptoms stemmed from first metatarsophalangeal joint arthritis, not pain from the fibrous dysplasia itself. The use of a stiff, hard-soled shoe aimed to prevent excessive motion at the joint, in combination with range-of-motion exercises and gait retraining. Nevertheless, a few years later, the pain in her first toe metatarsophalangeal joint progressed, becoming unbearable, causing walking difficulties and adversely affecting her quality of life.

It was clear through clinical and radiological examination that the first metatarsophalangeal arthritis is the source of pain and not the fibrous dysplasia of the first metatarsal. The pain was present on weight bearing and movements of the joint with no obvious history of trauma. The treatment options that were offered to the patient ranged from conservative to surgical. The conservative management was given for 3 years including orthotics, physiotherapy, analgesia and intra-articular injection. Injections were not required as a diagnostic modality however this option was offered to the patient as a conservative management option. We then discussed surgical management with the patient when there was failure of response to conservative therapy. This was either a fusion of the arthritic joint or to consider an arthroplasty to preserve range of motion. The patient preferred to preserve her range of MTP joint motion as opposed to having the joint fused. After considering all the options, she decided to proceed with informed consent for MTP joint arthroplasty after understanding the high risk of loosening and peri-prosthetic fracture.

**Figure 2:**
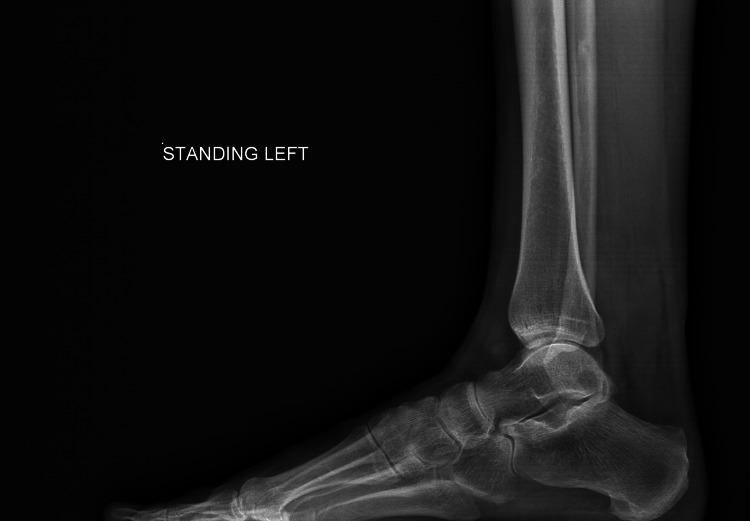
Lateral pre-operative X-ray showing arthritic changes in the left metatarsophalangeal (MTP) joint.

The patient underwent first metatarsophalangeal joint replacement (Figure 2) as a day case surgery with an uncomplicated recovery. The procedure was done using a general anaesthetic, ankle block and using standard surgical technique. This achieved a very good fixation on table along with excellent range of motion. The implant used is an uncemented prosthesis. The fixation on table is with tight fit off the canal on either side of the joint and there is eventual ongrowth of bone over the prosthesis stem. The ligaments were found to be stable and no sesamoidectomy was performed.

**Figure 3:**
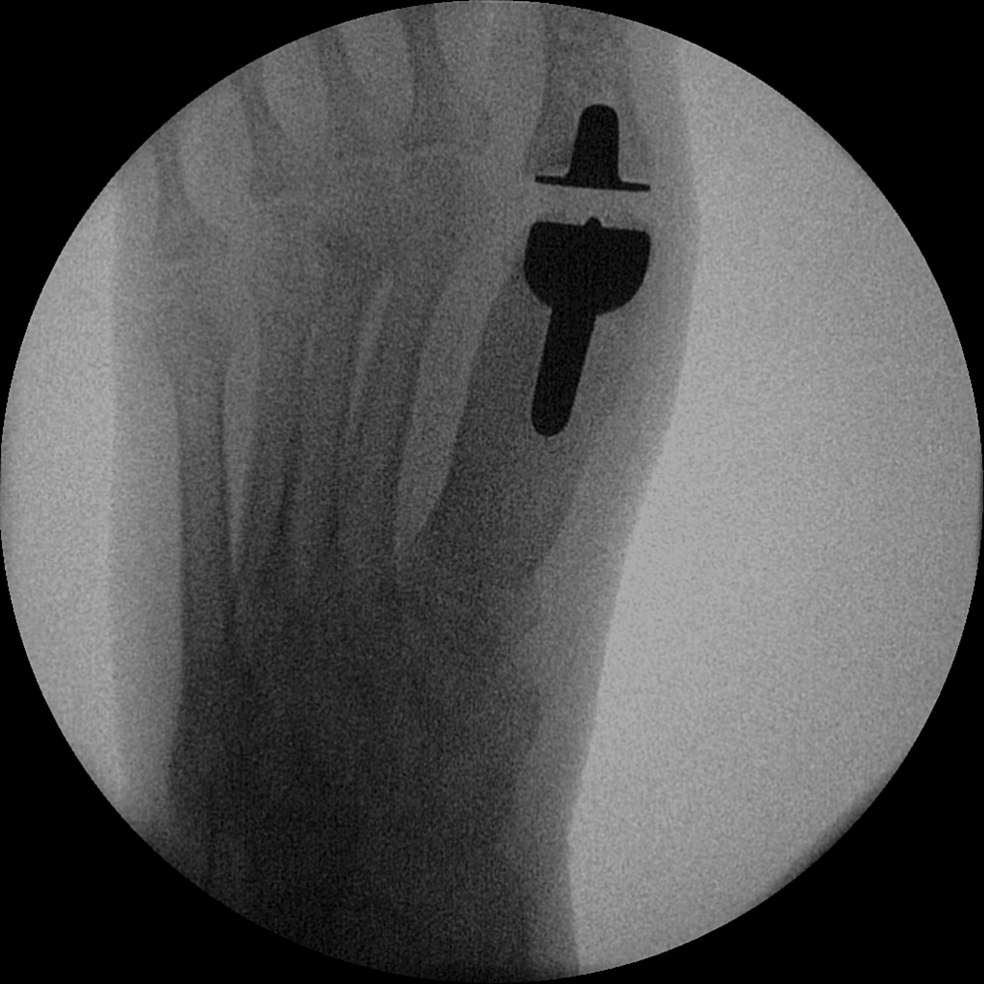
Intra-operative X-rays during first metatarsophalangeal joint replacement.

**Figure 4:**
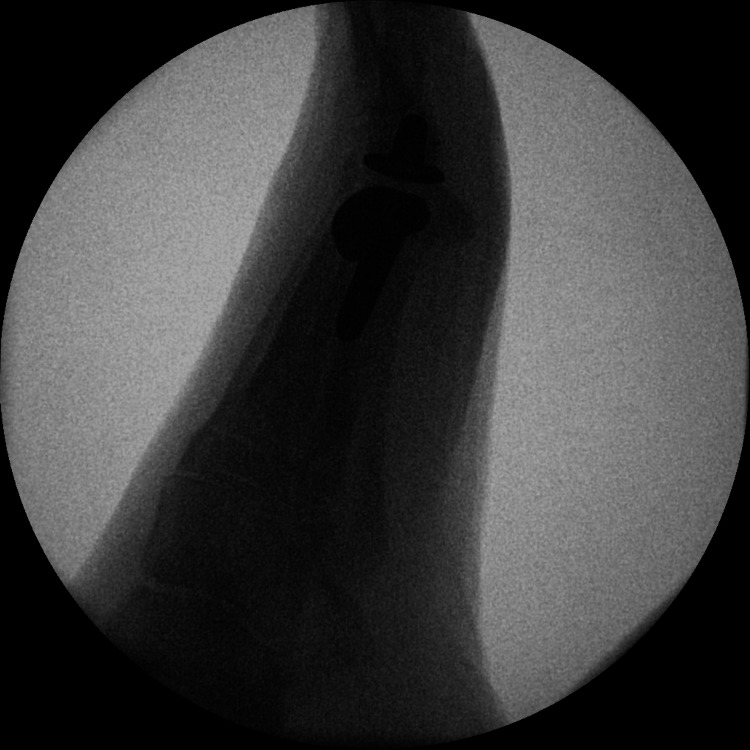
Intra-operative X-rays during first metatarsophalangeal joint replacement.

**Figure 5:**
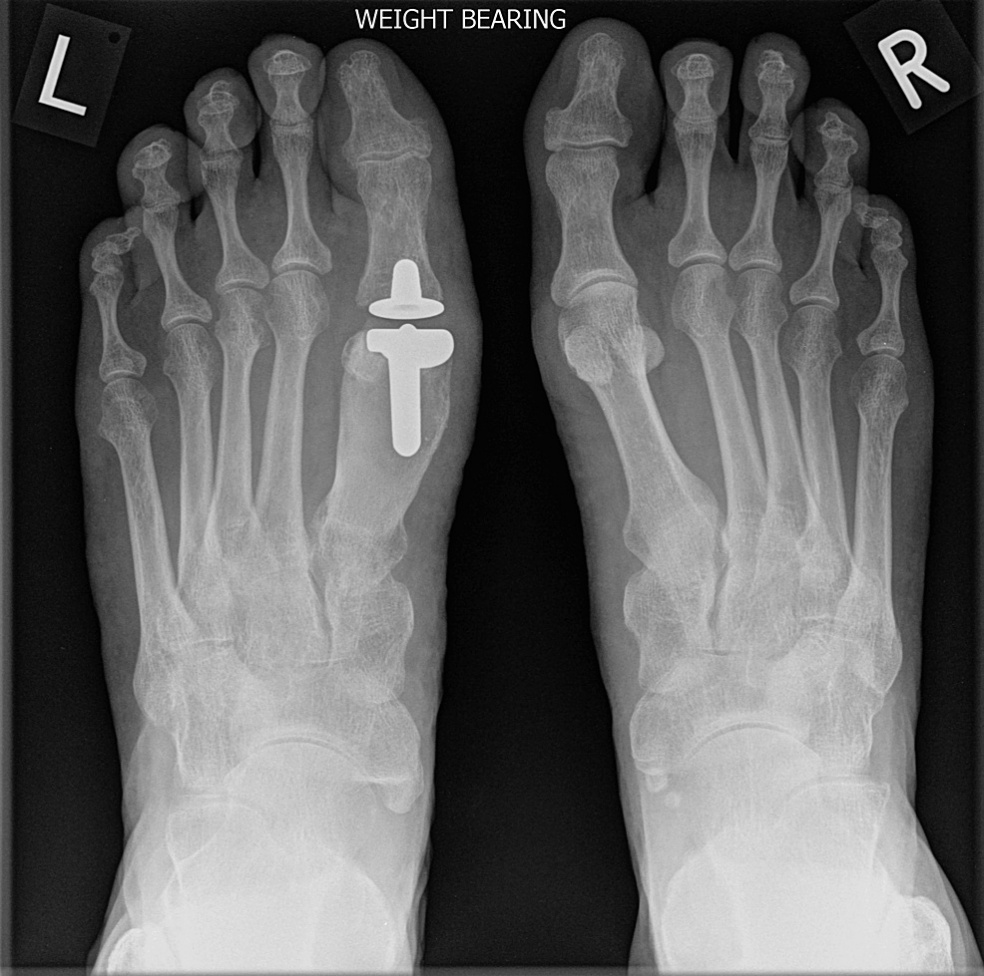
Anteroposterior post-operative X-ray at 36 months following first metatarsophalangeal joint replacement.

**Figure 6:**
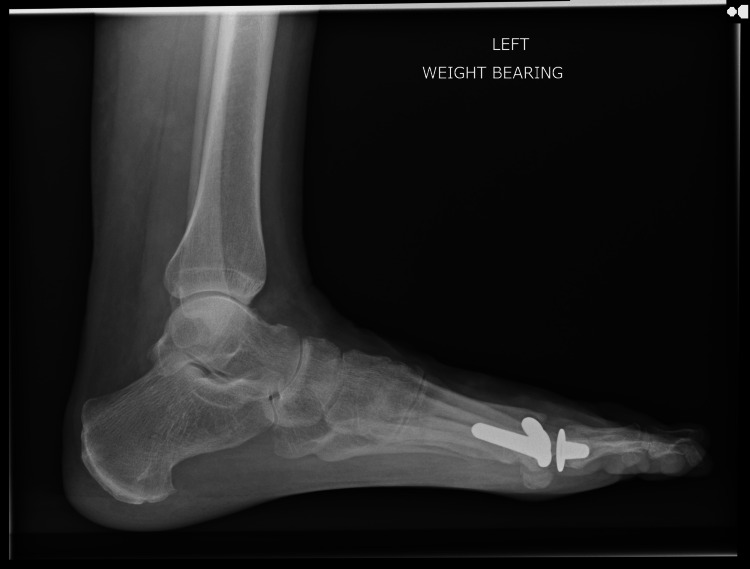
Lateral post-operative X-ray at 36 months following first metatarsophalangeal joint replacement.

Post-operatively, she was given a heel weight-bearing shoe for six weeks and then mobilized freely thereafter. A post-operative X-ray was taken at six weeks. Following the removal of sutures at two weeks, the patient was advised full weight bearing and was referred to physiotherapy for gaining further range of motion. The physiotherapy continued until a satisfactory range of motion was achieved. A final X-ray was taken at 36 months postoperatively, which revealed satisfactory osseointegration of the implant with no signs of loosening, osteolysis, or implant migration (Figures 3-6).

A pre-operative AOFAS sheet was completed, which showed 28 points, which increased to 88 post-operatively, confirming an excellent outcome. At 36 months follow-up, the patient responded very happy with the outcome. She has reported resolution in swelling, can walk around comfortably, and has 90° toe dorsiflexion and about 30° of plantarflexion. She is being followed up in the orthopaedic clinic annually. 

## Discussion

FD can present as monostotic, polyostotic disease, or craniofacial FD [1]. Most commonly, the long bones of the legs, ribs, face (posterior maxilla), and skull are affected [2]. Rarely affected regions include the clavicles, cervical and lumbar spine. Males and females are equally affected. Most patients are diagnosed during the first three decades of life, with children and adolescents being more frequently diagnosed. Pregnancy can aggravate the symptoms due to the estrogen receptors found in FD [2]. Small monostotic lesions are typically asymptomatic incidental findings and do not require any treatment. However, if symptomatic, complaints will include pain, swelling, and tenderness of the affected joint(s) and may require intervention [9]. Polyostotic disease can occur as part of a syndrome, e.g., McCune-Albright syndrome or Mazabraud syndrome, or in association with other endocrine dysfunctions, e.g., diabetes mellitus, hyperthyroidism or hyperparathyroidism [2]. Classically, FD lesions are intramedullary, expansile, and well-defined. Although endosteal scalloping may be present, a smooth cortical contour is always maintained. They exhibit hazy density with a ground-glass appearance. The lesions usually show nonspecific increased uptake of radiotracer on bone scans. MRI components of FD are variable, typically showing signal intensity that is intermediate to low on T1-weighted images and heterogeneous enhancement after administration of Gadolinium [9]. The gross appearance of FD is a firm, solid white mass replacing the medullary cavity. Typical microscopic findings include irregular spindles of woven bone, usually nonmineralized, scattered throughout a fibrocellular matrix. Foci of cartilage may also be present, sometimes leading to the potentially devastating misdiagnosis of chondrosarcoma. The degree of haziness shown radiographically by a given FD lesion directly correlates with its underlying histopathology [10].

Monostotic FD of the metatarsals and toes is a rare finding. There have been reports about this condition affecting the foot and third toe [4,5,6]. Previous papers have described the treatment of the symptoms with resection, amputation, or the use of free vascular fibular grafts [4,5,6]. Since the most common bone affected by FD is the proximal femur, hip replacements have been described for FD. However, there is a high incidence of loosening and revising femoral components [11,12]. This appears to be the first paper in the literature describing the use of the ROTOglide™ system for the first MTP joint arthroplasty in this condition. The ROTOglide™ system, consisting of a commercially pure titanium-coated and hydroxyapatite system, allows for normal joint flexibility. It comprises three parts: a metatarsal implant, metatarsal head, and an anatomical flange (phalangeal component). A polymeniscus is inserted between the metal pieces. Extension and flexion occur at the meniscus and metatarsal implant, while rotation takes place between the meniscus and metatarsal implant. The system aims to allow the patient to continue with their daily activities without the symptoms of osteoarthritis [7,8]. Richter M [7] and Karpe P et al. [8] have both published satisfactory outcomes with the ROTOglide™ first MTP joint replacement. Although there is a high risk of metatarsal prosthesis loosening, this patient's case has thus far had a good outcome without any loosening. Our post-operative results are satisfying so far, with the patient being very happy with the outcome of the surgery and managing her daily activities pain-free.

## Conclusions

This case report has discussed a patient with monostotic FD affecting the first metatarsal, leading to osteoarthritis in the first MTP joint, which impacted quality of life by causing significant pain, swelling, and walking difficulty. Following the first MTP joint replacement, there was an improvement in the American Orthopedic Foot and Ankle Score (AOFAS) (Pre-op: 28 points; Post-op: 88 points). This improvement, along with high patient satisfaction, a return to normal activity, and enhancements in pain management and range of motion, has confirmed excellent outcomes at the early stages of follow-up. Further experiences with longer-duration follow-ups would be beneficial to understand more thoroughly the indications and benefits of this procedure in monostotic FD.

## References

[1] Schoenau E, Rauch F (2002). Fibrous dysplasia. Horm Res.

[2] Fibrous Dysplasia (2017a (2020). Fibrous Dysplasia. https://rarediseases.org/rare-diseases/fibrous-dysplasia/.

[3] Tafti D, Cecava ND (2023). Fibrous Dysplasia. https://www.ncbi.nlm.nih.gov/books/NBK532947/.

[4] Dastgir N, Corrigan J, Kelly IP (2003). Fibrous dysplasia of a third toe. The Foot.

[5] Duncan GS (1987). Monostotic fibrous dysplasia of the foot. J Foot Surg.

[6] Tomczak RL, Johnson RE, Hamilton J (1993). The treatment of monostotic fibrous dysplasia of the first metatarsal with free vascularized fibular bone graft. J Foot Ankle Surg.

[7] Richter M (2019). Total joint replacement of the first metatarsophalangeal joint with Roto-Glide as alternative to arthrodesis. Fuß & Sprunggelenk.

[8] Karpe P, Killen MC, Chauhan A, Pollock R, Limaye R (2018). Early results of Roto-glide joint arthroplasty for treatment of hallux rigidus. Foot (Edinb).

[9] Stanton RP, Ippolito E, Springfield D, Lindaman L, Wientroub S, Leet A (2012). The surgical management of fibrous dysplasia of bone. Orphanet J Rare Dis.

[10] Riminucci M, Liu B, Corsi A, Shenker A, Spiegel AM, Robey PG, Bianco P (1999). The histopathology of fibrous dysplasia of bone in patients with activating mutations of the Gs alpha gene: site-specific patterns and recurrent histological hallmarks. J Pathol.

[11] Vaishya R, Singh AP (2010). Total hip replacement of recurrent monostotic fibrous dysplasia of proximal hip. J Clin Orthop Trauma.

[12] Sierra RJ, Cabanela ME (2009). Total hip arthroplasty in patients with underlying fibrous dysplasia. Orthopedics.

